# Cross-validation for training and testing co-occurrence network inference algorithms

**DOI:** 10.1186/s12859-025-06083-7

**Published:** 2025-03-06

**Authors:** Daniel Agyapong, Jeffrey Ryan Propster, Jane Marks, Toby Dylan Hocking

**Affiliations:** 1 School of Informatics, Computing, and Cyber Systems, Northern Arizona University, Flagstaff, AZ USA; 2 Department of Biological Sciences, Northern Arizona University, Flagstaff, AZ USA; 3 Département d’informatique, Université de Sherbrooke, Sherbrooke, Canada

**Keywords:** Co-occurrence network inference, Machine learning, Cross-Validation, LASSO, Microbiome Analysis, Network Validation, Compositional Data, High-dimensional Statistics, Ecological Networks

## Abstract

****Background**:**

Microorganisms are found in almost every environment, including soil, water, air and inside other organisms, such as animals and plants. While some microorganisms cause diseases, most of them help in biological processes such as decomposition, fermentation and nutrient cycling. Much research has been conducted on the study of microbial communities in various environments and how their interactions and relationships can provide insight into various diseases. Co-occurrence network inference algorithms help us understand the complex associations of micro-organisms, especially bacteria. Existing network inference algorithms employ techniques such as correlation, regularized linear regression, and conditional dependence, which have different hyper-parameters that determine the sparsity of the network. These complex microbial communities form intricate ecological networks that are fundamental to ecosystem functioning and host health. Understanding these networks is crucial for developing targeted interventions in both environmental and clinical settings. The emergence of high-throughput sequencing technologies has generated unprecedented amounts of microbiome data, necessitating robust computational methods for network inference and validation.

****Results**:**

Previous methods for evaluating the quality of the inferred network include using external data, and network consistency across sub-samples, both of which have several drawbacks that limit their applicability in real microbiome composition data sets. We propose a novel cross-validation method to evaluate co-occurrence network inference algorithms, and new methods for applying existing algorithms to predict on test data. Our method demonstrates superior performance in handling compositional data and addressing the challenges of high dimensionality and sparsity inherent in real microbiome datasets. The proposed framework also provides robust estimates of network stability.

****Conclusions**:**

Our empirical study shows that the proposed cross-validation method is useful for hyper-parameter selection (training) and comparing the quality of inferred networks between different algorithms (testing). This advancement represents a significant step forward in microbiome network analysis, providing researchers with a reliable tool for understanding complex microbial interactions. The method’s applicability extends beyond microbiome studies to other fields where network inference from high-dimensional compositional data is crucial, such as gene regulatory networks and ecological food webs. Our framework establishes a new standard for validation in network inference, potentially accelerating discoveries in microbial ecology and human health.

## Background

Microorganisms form complex ecological interactions such as mutualism, parasitism/predation, competition, commensalism and amensalism [1]. The human body hosts complex microbial communities consisting of bacteria, protozoa, archaea, viruses, and fungi. The human intestine alone has trillions of bacteria (microbiota), that have a symbiotic relationship with the host. The main function of the microbiota is to protect the intestine against colonization by harmful microorganisms like pathogens through mechanisms, such as competition for nutrients and modulation of host immune responses. Studying the interaction of the microbiota with pathogens and the host can offer new insights into disease pathogenesis and potential treatments [2]. Over the past several years, the importance of the microbiome to human health and disease has become increasingly recognized. The trillions of microbes can protect us from colonization by pathogens, promote immunoregulation and tolerance by our own immune systems, and digest many of the foods that we ourselves cannot [3]. However, they can also contribute to disease, if their balance is disrupted by antibiotics, immune dysregulation, or other disturbances. The focus of this field has largely been on the bacterial members of the microbiome, since they make up the largest proportion of microbiota [4]. The bacteria exist alongside a diversity of organisms which can interact with each other and the host to impact health [5]. Therefore, understanding the ecological interactions that occur in microbial communities is very crucial in maintaining a well-functioning ecosystem [6]. To understand the interactions of microbial communities, it is beneficial to construct ecological networks that depict their positive and negative associations [7]. These networks, known as co-occurrence networks, have become an essential tool in microbial ecology and biomedical research [8]. Co-occurrence networks are graphical representations where nodes represent microbial taxa, and edges represent significant associations between taxa [9]. These associations can be positive (indicating potential cooperation or similar environmental preferences) or negative (suggesting competition or antagonism) [6]. Co-occurrence networks help researchers visualize and understand complex microbial ecosystems, revealing key players and their relationships [10]. In medical microbiology, these networks can highlight differences between healthy and diseased states, potentially identifying microbial signatures of various conditions [8]. Ecologists use co-occurrence networks to study how microbial communities respond to environmental changes, crucial for understanding climate change impacts [11]. In soil and plant microbiome studies, these networks help identify beneficial microbial associations that could improve crop yields [12]. The field of microbial co-occurrence networks has been significantly advanced by the contributions of numerous researchers and institutions. Jed Fuhrman and colleagues at the University of Southern California have been pioneers in applying network analysis to marine microbial ecology. Their work has been instrumental in revealing complex interactions among marine microbes and their responses to environmental changes [13]. In the field of human microbiome research, Rob Knight and his team at the University of California, San Diego have made substantial contributions. Their application of network analysis to human microbiome studies has revealed intricate relationships between different microbial taxa and their associations with human health and disease [14]. This work has been crucial in advancing our understanding of how microbial communities influence human physiology and pathology. Janet Jansson’s group at the Pacific Northwest National Laboratory has been at the forefront of applying co-occurrence networks to soil microbiomes. Their research has elucidated how soil microbial communities respond to various environmental factors, including climate change and agricultural practices [15]. These studies have important implications for sustainable agriculture and ecosystem management in the face of global environmental changes. Numerous algorithms exist for inferring these networks, each with their own set of hyper-parameters used to determine the level of sparsity (number of edges) in a network. The choice of algorithm and the tuning of these parameters can significantly impact the resulting network structure and, consequently, the biological interpretations drawn from it. Several comprehensive reviews have examined different aspects of microbial network inference. For instance, Kurtz et al. (2023) [16] conducted a systematic evaluation of network inference methods from amplicon data, providing valuable insights into the strengths and limitations of various approaches. Similarly, Zhang and Sun (2024) [17] reviewed current modeling techniques and tools for studying microbial interactions, while Liu et al. (2021) [18] presented a mini-review focusing specifically on network analysis methods for microbial communities. These studies collectively highlight the evolving nature of the field and the importance of choosing appropriate methods for specific research contexts.

### Microbiome composition data sets

**Fig. 1:**
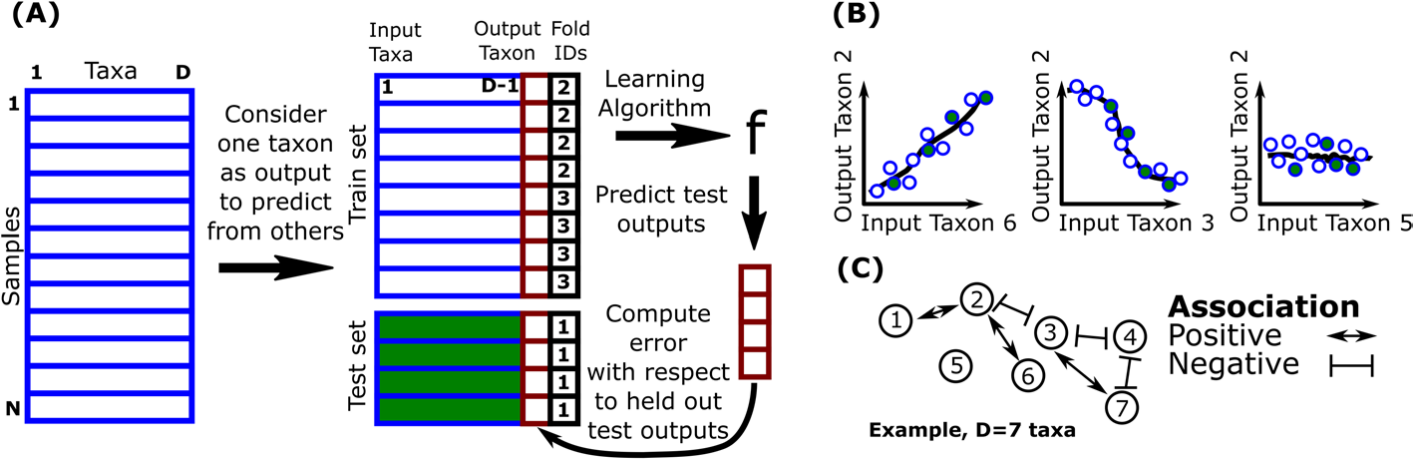
**A** Proposed cross-validation for evaluating network inference algorithms.** B** Learned regression model.** C** Co-occurrence network: Nodes represent distinct taxa/bacteria and edges represent positive or negative associations

There have been some challenges in obtaining microbiome abundance in different environments [19]. High-throughput Sequencing is used to sequence large amounts of DNA fragments at relatively low cost [20]. This involves amplifying a particular region of the bacterial genome through Polymerase Chain Reaction (PCR) and subsequently sequencing the produced amplicons. This region represents the 16S rRNA gene in bacteria, extensively used as indicators for microbial classification and identification. The processed sequences are classified into Operational Taxonomic Units (OTU) with the aid of an advanced software that compares the sequences to a reference database such as the Ribosomal Database Project [21] and the Green Genes Database [22]. Table 1 presents some real microbiome composition data from public sources. Each Operational Taxonomic Unit (OTU) data describes the taxonomic composition of different samples from various environments. The percentage of zero entries in the data is displayed in the sparsity column. In Fig. 1, the microbiome composition data set is represented by a matrix $N \times D$ of counts (abundance) of bacteria, where each column represents a different type of bacteria (taxon) and each row represents a different sample.

**Table 1:** Publicly available microbiome composition datasets

Data	Algorithm	Samples	Taxa	Sparsity (%)
glne007	mLDM [23]	490	338	58.88
Baxter_CRC	mLDM [23]	490	117	27.78
amgut2	SPIEC_EASI [24]	296	138	34.60
amgut1	SPIEC_EASI [24]	289	127	30.40
enterotype	phyloseq [25]	280	553	67.62
MixMPLN_real_data	MixMPLN [26]	195	129	69.82
crohns	MDiNE [27]	100	5	1.00
iOraldat	COZINE [28]	86	63	43.10
soilrep	phyloseq [25]	56	16825	69.82
hmp216S	SPIEC_EASI [24]	47	45	12.67
hmp2prot	SPIEC_EASI [24]	47	43	14.05
esophagus	phyloseq [25]	58	3	43.10

### Categorization of previous algorithms

Many algorithms have been proposed to infer co-occurrence networks from real microbiome data sets. In Table 2, we group previous network inference algorithms into four categories: Pearson correlation (Pearson), Spearman correlation (Spearman), Least Absolute Shrinkage and Selection Operator (LASSO), and Gaussian Graphical Model (GGM).

For example, SparCC [29] estimates the Pearson correlations of log-transformed abundance data and uses an arbitrary threshold to limit the network, whereas MENAP [30] uses Random Matrix Theory to determine the correlation threshold of the standardized relative abundance data. MANIEA [31], improve the basic co-occurrence methods by incorporating environmental adaptation factors directly into the model.

Both CCLasso [32] and REBACCA [33] employ LASSO to infer correlations among microbes using log-ratio transformed relative abundance data. MAGMA [34] employs L1 penalty to enforce sparsity in the estimation of the precision matrix.

The field has seen substantial development in GGM-based approaches. Early methods like mLDM [23] and SPIEC-EASI [24] introduced basic graphical models, with mLDM focusing on microbe-environment associations and SPIEC-EASI on conditional dependencies between microbes. MicroNet-MIMRF [36] uses mixed integer optimization for network inference whereas SPLANG [36] further extends these approaches by incorporating gene regulatory information into the network inference process.

There are other algorithms, such as Mutual Information (MI), which can capture both linear and nonlinear associations between microbial species. Unlike traditional correlation metrics, which are limited to linear relationships, MI is capable of detecting a wider range of dependencies by measuring the amount of shared information between two variables. This makes it particularly useful in microbiome studies, where interactions between species are often complex and may not follow simple linear patterns. Techniques such as ARACNE [37] and CoNet [38] utilize MI to construct microbial co-occurrence networks. These tools often employ additional steps, like the Data Processing Inequality (DPI), to filter out indirect associations, reducing the likelihood of false positives and improving the accuracy of the inferred networks [39]. The implementation of MI is straightforward as the scikit-learn [40] library provides efficient functions for its calculation. However, for our cross-validation approach, the conditional expectation required is mathematically complex and not easily defined. This complexity arises because the calculation of conditional expectation in the context of MI often involves estimating joint distributions of multiple variables, which becomes difficult to apply, particularly in high-dimensional microbiome datasets.

**Table 2:** Categories of microbial network inference algorithms

Category	Notable methods
Pearson	SparCC (2012) [29], MENAP (2012) [30], CoNet (2016) [41], MANIEA (2021) [31]
Spearman	MENAP (2012) [30], CoNet (2016) [41]
LASSO	CCLasso (2015) [32], REBACCA (2015) [33], SPIEC-EASI (2015) [24], MAGMA (2019) [34]
GGM	REBACCA (2015) [33], SPIEC-EASI (2015) [24], gCoda (2017) [42], mLDM (2020) [23], MDiNE (2019) [27], HARMONIES (2020) [43], COZINE (2020) [28], PLNmodel (2021) [44], Multinomial VA (2021) [45], SPLANG (2024) [36], MicroNet-MIMRF (2024) [36]

### Previous sparsity hyper-parameter training methods

Hyper-parameters are configuration variables that are external to the model and whose values cannot be estimated from the data; they are often set before the learning process begins and significantly influence the model’s performance. Each of the algorithms have their own set of hyper-parameters used to determine the level of sparsity (number of edges) in a co-occurrence network. For instance, in the Pearson and Spearman correlation inference algorithms, there is a threshold on the correlation coefficient which is typically chosen arbitrarily or using prior knowledge [29, 30, 41]; edges with absolute coefficient magnitude below the threshold are removed from the network. The LASSO uses the degree of L1 regularization, typically selected using cross-validation to determine the sparsity of the network [32]. The GGM infers the conditional dependencies between taxa by estimating the sparsity pattern of the precision matrix using penalized maximum likelihood methods through cross-validation [24].

### Previous evaluation criteria

Various evaluation criteria have been utilized to assess the performance of different algorithms used for network inference. The most common approaches can be categorized into three main types: external data validation, network consistency analysis, and synthetic data evaluation. External data validation, used by early methods like SparCC [29] and SPIEC-EASI [24], is based on the comparison of inferred networks with known biological interactions. However, this approach is limited by the scarcity of reliable ground-truth data and potential biases in external datasets. Network consistency analysis, exemplified by CCLasso [32], evaluates the stability of inferred networks across different subsamples. Although this approach helps assess reproducibility, it may favor overly sparse networks that show perfect consistency by inferring few or no edges. Our cross-validation framework addresses this limitation by focusing on prediction accuracy rather than mere consistency, ensuring that inferred relationships generalize meaningfully to new data. Recent methods have increasingly employed synthetic data evaluation. For instance, MANIEA [31] uses ROC curves on simulated networks, while MicroNet-MIMRF [36] employs edge recovery rates on synthetic data. SPLANG [36] combines multiple metrics including F1 scores and modularity measures. While synthetic evaluation offers controlled testing environments, it may not fully capture the complexity of real biological networks. Table 3 provides a comprehensive overview of evaluation approaches used by different algorithms, including their comparison methods and specific evaluation metrics. The diversity of evaluation criteria underscores the challenge of establishing standardized performance assessment in microbial network inference.

**Table 3:** Existing evaluation methods

Algorithm	Algorithms compared	How they compare	Evaluation type
SparCC(2012)	SparCC, Pearson	Confusion matrix detected in the Pearson network by treating the SparCC network as the ground truth	External data (HMPOC dataset, build 1.0) [46]
REBACCA(2015)	REBACCA, SparCC, BP, ReBoot	Consistency of positive and negative correlated taxonomic pairs identified independently from three data sets	External data (Mouse skin microbiota) [47]
SPIEC-EASI(2015)	SPIEC-EASI, SparCC, CCREPE	Consistency of sub-samples by measuring the Hamming distance between a reference network and inferred network	External data (American Gut data set) [48]
CCLasso(2015)	CCLasso, SparCC	Frobenius accuracy between the estimated correlation matrices and a reference correlation matrix from data using half samples	Sub-sample analysis
gCoda(2017)	gCoda, SPIEC-EASI	False positive count on shuffled OTU data	External data (Mouse Skin microbiome data) [47]
MAGMA(2019)	MAGMA, SPIEC-EASI, gCoda	Graph accuracy evaluation using precision-recall curves and network sparsity patterns	Synthetic data and TARA ocean data
HARMONIES(2020)	HARMONIES, SPIEC-EASI, CCLasso, Pearson	Accuracy of identifying true positive edges by comparing the estimated precision matrix with an arbitrarily chosen true one	External data
mLDM(2020)	mLDM, SparCC, CCLasso	Power of association inference when compared to the reference association inference data [49]	External data (Tara Oceans Eukaryotic data)
COZINE(2020)	COZINE, SPIEC-EASI, Ising	The assortativity coefficient [50] (The likelihood of taxa existing within the same branch of the taxonomic tree to be interconnected within co-occurrence networks)	External data (Oral microbiome data)
MANIEA(2021)	MANIEA, SPIEC-EASI, SparCC, REBACCA	Network inference accuracy using ROC curves and environmental adaptation scores	Synthetic data and human gut microbiome
Multinomial VA(2021)	Multinomial VA, SPIEC-EASI, gCoda	Edge detection accuracy using precision matrices and stability selection	Synthetic data and American Gut Project
SPLANG(2024)	SPLANG, SPIEC-EASI, MANIEA	Network structure accuracy using F1 scores and modularity metrics	Synthetic data and gut microbiome
MicroNet-MIMRF(2024)	MicroNet-MIMRF, SPIEC-EASI, gCoda	Edge recovery rates and false discovery control using mixed integer optimization	Synthetic and TARA ocean data

**Table 4:** Summary of contributions

Algorithm	Cross-validation for training	Cross-validation for testing
LASSO	CCLasso (2015) [32], REBACCA (2015) [33], SPIEC-EASI (2015) [24]	Proposed
GGM	gCoda (2017) [42], MDiNE (2019) [27], COZINE (2020) [28]	Proposed
Correlation (Pearson/Spearman)	Proposed	Proposed

**Summary of contributions**

Table 4 summarizes the key contributions of our paper, which extend and enhance existing methodologies for inferring and evaluating microbial co-occurrence networks. The table is organized by algorithm type and distinguishes between cross-validation methods used for training and those proposed for testing. In the category of LASSO (Least Absolute Shrinkage and Selection Operator) algorithms, several existing methods have utilized cross-validation for training. CCLasso, introduced by Fang et al. [51], employs cross-validation to optimize the regularization parameter in their compositional data analysis approach. Similarly, REBACCA [52] and SPIEC-EASI [53] use cross-validation in their respective LASSO-based approaches to infer microbial associations. Our contribution in this category is the proposal of a novel cross-validation method for testing LASSO-based models, which addresses a gap in the existing methodologies and potentially improves the robustness of network inferences. For Gaussian Graphical Models (GGM), several algorithms have previously incorporated cross-validation in their training processes. The gCoda method, developed by Fang et al. [54], uses cross-validation to select optimal parameters for their graphical model. MDiNE [55] and COZINE [28] also employ cross-validation in their GGM-based approaches to microbial network inference. Our contribution extends these methods by proposing a new approach for using cross-validation to test GGM-based models. In the context of correlation-based methods (Pearson and Spearman), our paper introduces novel approaches for both training and testing using cross-validation. This represents a significant advancement over previous methods, which often relied on arbitrary thresholds or prior knowledge to determine significant correlations. By introducing cross-validation to both the training and testing phases of correlation-based network inference, we aim to enhance the reliability and reproducibility of these widely-used methods. In this paper, we present novel contributions that extend the existing research in this field. Firstly, we introduce new techniques for leveraging well-established algorithms such as Pearson/Spearman correlation and Gaussian Graphical Model for prediction on held-out or test data. Secondly, we propose the utilization of prediction error on test set in cross-validation as a more widely applicable method for evaluating various algorithms on real microbiome data. Lastly, we propose training the optimum correlation threshold in correlation based algorithms with cross-validation as compared to previous methods that use prior knowledge or pre-determined correlation threshold. Although several new methods have been developed recently (MANIEA [31], SPLANG [36], MicroNet-MIMRF [36]), none of these utilize cross-validation for testing, further highlighting the novelty and potential impact of our proposed testing methodology.

## Methods

### Preprocessing and normalization of dataset

Microbial data sets are very high-dimensional in nature because they have substantial number of taxa that can be present in a single sample [56]. Their sparse nature makes even conventional machine learning algorithms struggle since they assume that most features are non-zero [57]. Hence, it is crucial to apply appropriate preprocessing and normalization technique to convert the data set to a suitable format before conducting any data analysis [58]. These are some of the notable methods for transforming sparse microbial data sets.

#### Standard scaling

Standard scaling normalizes each taxon column to have zero mean and unit variance for numerical stability. This can help to reduce the influence of outliers and scale differences among taxa. Let *N* be the sample size, $\bar{x_j}$ be the mean of the $j^{th}$ taxa across all samples, $s_j$ be the standard deviation of the $j^{th}$ taxa across all samples and $x_{ij}$ be the count of taxon *j* in sample $i\in \{1,\dots ,N\}$. The standard scaling transformation is given by:

$$\begin{aligned} x_{ij} = \frac{x_{ij} - \bar{x_j}}{s_j} \end{aligned}$$

#### Yeo-Johnson power transformation

The Yeo-Johnson power transformation is a method for transforming numerical variables to approximate a normal distribution [59]. This transformation is inspired by the log transformation that has been used in previous studies [29, 32, 33], but it differs in the mathematical function that it applies depending on the sign of the count value. Moreover, it involves a power parameter that determines the extent of the transformation and that is estimated from the data itself using the maximum likelihood method [59]. Let $\lambda$ be the power parameter, $x_{ij}$ be the count data and $y^{(\lambda )}$ be the transformed count data. The Yeo-Johnson transformation is given by:

$$\begin{aligned} x_{ij}^{(\lambda )} = {\left\{ \begin{array}{ll} \left[ \left( y+1\right) ^{\lambda }-1\right] / \lambda , & \text{ if } \lambda \ne 0, y \ge 0 \\ \log \left( y+1\right) , & \text{ if } \lambda = 0, y \ge 0 \\ -\left[ \left( -y+1\right) ^{2-\lambda }-1\right] / (2-\lambda ), & \text{ if } 2-\lambda \ne 0, y< 0 \\ -\log \left( -y+1\right) , & \text{ if } 2-\lambda = 0, y < 0 \end{array}\right. } \end{aligned}$$

### Cross-validation for evaluating co-occurrence network inference algorithms

Cross-validation is a standard algorithm in machine learning used for selection, evaluation and estimation of performance of models. It has been previously used in the context of microbiome for training co-occurrence network inference algorithms [32]. Our study introduces cross-validation as a novel criterion to test the performance of co-occurrence network inference algorithms on microbiome data. In Fig. 1A, we show how $K=3$ fold cross-validation can be used in the context of microbiome data.

We used $K=3$ folds because our small sample size limits the ability to split the data into larger training and testing sets, which could reduce model stability. Choosing $K=3$ also reduces the computational burden by minimizing the number of training iterations compared to larger K values. While larger K values generally provide more stable results by using more data for training, they also increase computation time [60]. The analysis is repeated D times, each time using a different taxon as the outcome variable and the remaining taxa as the predictor variables. We randomly split the data into 3 folds. One of the three folds is used as test set whilst the other two folds are used as the train set. We fit each algorithm on the train set, which is further split into subtrain and validation sets to learn the hyper-parameters of the model. We select the best model based on the validation score and fit it on the whole training set. We then evaluate it on the test set. We repeat this process 3 times and average the test errors to get the overall performance metric. Our Mean Squared Error calculations validate prediction accuracy on held-out abundance data, rather than validating the inferred interactions themselves against ground truth networks. Although successful prediction suggests that the model has captured meaningful relationships, direct validation of microbial interactions would require experimental verification, which is not always available.

We show a learned regression model in Fig. 1B from cross-validation which is used to infer the co-occurrence network in Fig. 1C. As shown in the network graph where $D=7$ taxa, there is an edge between two taxa only if the relationship between them is positive or negative.

### Correlation based methods

#### Pearson correlation

Pearson correlation coefficient is the standard tool to infer a network through correlation analysis among all pairs of OTU (Operational Taxonomic Unit) samples. It measures the strength and direction of the relationship between two variables. It ranges from −1 to 1, where −1 indicates a perfect negative linear relationships, 0 indicates no linear relationship and +1 indicates a perfect positive relationship. In most literature [29, 30, 41], there is an arbitrary or pre-determined threshold chosen to select the range of values which is regarded as proof of positive or negative association. For a pair $(x_1,x_2)$ of standard scaled taxa that follow a bivariate normal distribution with Pearson correlation coefficient $\rho _{x_1,x_2}$, marginal standard deviations $\sigma _{x_1}$ and $\sigma _{x_2}$, the predicted value of $x_1$ given $x_2$ is given below.

1 $$\begin{aligned} x_1 = \rho _{x_1,x_2}\frac{\sigma _{x_1}}{\sigma _{x_2}}(x_2) \end{aligned}$$

This expression is used to compute a prediction for the test set given a trained model that was fit on a training set. The parameter, $\rho _{x_1,x_2}$ is learned from the training data.

#### Spearman correlation

Spearman correlation coefficient is another popular correlation method for microbial network inference. It is often adopted as an alternative to the Pearson correlation coefficient when dealing with non-linear relationships between taxa. It is less sensitive and robust to outliers. Just as Pearson coefficient, the value of the Spearman coefficient ranges from −1 to +1 , with −1 indicating a perfect negative monotonic relationship, 0 indicating no monotonic relationship, and +1 indicating a perfect positive monotonic relationship. Spearman coefficient is the Pearson coefficient of ranked data [61]. We implemented the Spearman algorithm by converting the data into ranks adopting the Pearson Correlation algorithm to predict the ranks. For a pair $(r(x_1), r(x_2))$ of standard scaled taxa that follow a bivariate normal distribution with correlation coefficient $\rho _{x_1,x_2}$, marginal standard deviations $\sigma _{r(x_1)}$ and $\sigma _{r(x_2)}$, the predicted rank value, $r(x_1)$ given $r(x_2)$ is given below.

2 $$\begin{aligned} r(x_1) = s_{x_1,x_2}\frac{\sigma _{r(x_1)}}{\sigma _{r(x_2)}}(r(x_2)) \end{aligned}$$

The model contains the parameters $\sigma _{r(X)}$, $\sigma _{r(Y)}$ and $s_{x_1,x_2}$, which are estimated from the training data. We used linear interpolation [62] to infer the actual predicted values from the predicted ranks. Linear interpolation is a technique widely adopted to estimate a value within a range of known values by calculating the proportionate relationship between the known values. Therefore, we utilize the actual values of the training data alongside their corresponding ranks to estimate the real values of the predicted ranks for the test data. Specifically, we use the known pairs of (value, rank) in the training data to form a linear relationship between values and their ranks. We then apply this relationship to the predicted ranks to estimate the actual values.

#### Least absolute shrinkage and selection operator (LASSO)

The LASSO is a form of linear regression which uses L1 regularization technique and taxon selection to increase the accuracy of prediction [63]. L1 regularization adds a penalty which causes the regression coefficient of the less contributing taxon to shrink to zero or near zero. In this algorithm, the overall objective is to minimize the loss function with respect to the coefficients. Let $\textbf{X} \in \mathbb {R}^{N \times D}$ be the compositional data matrix where each row represents a sample and each column represents a taxon, $\textbf{y} \in \mathbb {R}^n$ be the target taxon vector, $\textbf{w} \in \mathbb {R}^p$ be the coefficient vector, and $\beta _0$ be the intercept term. The linear model can be defined as:

3 $$\begin{aligned} f(\textbf{x}) = \beta _0 + \textbf{x}^T \textbf{w} \end{aligned}$$

Then, the loss function of the LASSO regression model can be formulated as:

$$\begin{aligned} L(\beta _0,\textbf{w}) = \frac{1}{2n}||\textbf{y} - \beta _0 - \textbf{X}\textbf{w}||^2_2 + \lambda ||\textbf{w}||_1 \end{aligned}$$

The first term is the residual sum of squares (RSS), which is the deviation of the predicted values from the actual values. The second term is the L1 penalty term that encourages sparsity in the coefficient estimates. $\lambda$ is the regularization parameter that controls the amount of shrinkage. With cross-validation algorithm, optimum LASSO model is selected and the coefficient of this model is used for network inference [40]. Train set is split into subtrain set (used to learn regression coefficients) and validation set (used to learn the degree of L1 regularization, which controls sparsity / number of edges in co-occurrence network).

### Gaussian graphical model (GGM)

The Gaussian distribution is a continuous and symmetrical probability distribution that explains how the outcomes of a random variable are distributed. The shape of the Gaussian distribution is determined by its mean and standard deviation, which evaluates the location and spread of the distribution, respectively. Most observations cluster around the mean of the distribution [64]. The Probability Density Function (PDF) of a multivariate normal distribution is frequently employed in data analysis to model complex data sets that involve multiple variables [65]. Let *D* be the total number of taxa, $\varvec{x}$ be a *D*-dimensional row/sample vector, $\varvec{\Sigma }$ be a $D\times D$ covariance matrix, $\varvec{\Omega }$ be a $D\times D$ precision matrix comprised of $\omega _{ij}$ elements and $\varvec{x}^T$ denote the transpose of $\varvec{x}$. The multivariate normal distribution PDF is given below.

$$\begin{aligned} f(\varvec{x}) = \frac{1}{\sqrt{(2\pi )^D|\varvec{\Sigma }|}}\exp \left( -\frac{1}{2}\varvec{x}^T\varvec{\Omega }\varvec{x}\right) \end{aligned}$$

The predicted value of the first taxon ($x_1$) can be calculated by finding the conditional mean of the distribution. This is the value of $x_1$ when *f*(*x*) is maximum. Therefore, we take the partial derivative of *f*(*x*) with respect to $x_1$ and equate to zero. As demonstrated in the Gaussian Graphical Model Proof, we solve for the value of $x_1$, which leads us to the following equation which we use to compute predictions,

4 $$\begin{aligned} x_1 = \frac{-1}{2\omega _{11}}\left( \sum _{i=2}^D \omega _{i1} x_i + \sum _{j=2}^D \omega _{1j} x_j \right) \end{aligned}$$

This is well known for the special case of $D=2$ (See proof in Supplementary Information), the conditional mean of a bivariate normal (1) under the assumption that data is standard scaled thus zero mean and unit variance. Our contribution here is to derive a formula for the general case, $D>2$ (See proof in Supplementary Information). The inverse covariance matrix (precision matrix) is computed from the train dataset in the GGM. The conditional independence structure among taxa is represented by the sparsity pattern of the precision matrix. This sparsity pattern can be estimated from data using various methods, such as maximum likelihood estimation or penalized likelihood methods. The Graphical Lasso (GLASSO) is used to estimate the precision matrix from high-dimensional data. In GLASSO, the penalty is applied to the elements of the precision matrix, resulting in a sparse estimate of the matrix. Given a train data matrix $\textbf{X} \in \mathbb {R}^{N \times D}$ where *N* is the number of samples and *D* is the number of taxa, the goal is to estimate the precision matrix $\varvec{\Theta }$ that satisfies the following optimization problem. Let $\textbf{S} = \frac{1}{N} \textbf{X}^\top \textbf{X}$ be the sample covariance matrix, $\Vert \varvec{\Theta } \Vert _1$ be the L1-norm penalty term to promote sparsity in the precision matrix, $\lambda$ be the regularization parameter that controls the strength of the penalty term. The precision matrix is given by:

$$\begin{aligned} \hat{\varvec{\Theta }} = \underset{\varvec{\Theta } \succeq 0}{\operatorname {argmin}} \left( \operatorname {tr}(\textbf{S}\varvec{\Theta }) - \log \det (\varvec{\Theta }) + \lambda \Vert \varvec{\Theta } \Vert _1 \right) \end{aligned}$$

The constraint $\varvec{\Theta } \succeq 0$ enforces the positive semi-definiteness of the precision matrix. The solution $\hat{\varvec{\Theta }}$ corresponds to the maximum likelihood estimate of the precision matrix under the sparsity constraint. The precision matrix is used to infer the network graph of the taxa based on their conditional dependencies. The presence or absence of an edge between taxa *i* and *j* in the graph is determined by the value of $\hat{\Theta }_{ij}$ in the precision matrix. An edge between taxa *i* and *j* exists if and only if $\hat{\Theta }_{ij} \ne 0$.

### Microbial association network inference

Following the identification of the optimal model for each algorithm, pairwise positive and negative associations between taxa in the data sets are computed to infer the co-occurrence network. For the correlation-based algorithms, the correlation matrix is estimated by calculating the pairwise correlation coefficient for each taxon pair, and the network is constrained by the learned correlation threshold. In the case of the LASSO algorithm, we save the coefficients of the optimal model at each iteration of the taxa columns, hence forming an association matrix. For the GGM, the GLASSO inferred precision matrix is used for the association matrix. We compute the mean of the upper and lower triangular matrices for each of the LASSO and the GGM, resulting in lower triangular matrices for each algorithm. In the resultant lower triangular matrix of the association matrix, an edge is identified if its value is non-zero. A positive value indicates a positive association, while a negative value indicates a negative association. Edge probabilities can be estimated by averaging the presence of an edge across the different folds, providing a measure of confidence for each inferred edge. Through the application of 3-fold cross-validation analysis, three networks are inferred for each algorithm based on the fold IDs. The final network obtained is the median of the three networks inferred by the 3 folds. While different algorithms employ varying criteria for determining significant interactions, our cross-validation framework provides a unified approach for evaluating these criteria. The prediction error on the test set serves as a unified metric across all algorithms, allowing for objective comparison of different significance thresholds. This addresses a key challenge in the field where different methods use distinct criteria for edge detection. Our approach suggests that optimal thresholds can be determined by minimizing prediction error, providing a data-driven way to identify significant interactions.

## Results and discussions

In this study, we conducted a real microbiome composition data analysis on Amgut [24], crohns [27] and iOral [28] data sets because they are public and widely used when comparing previous algorithms. We wrote python code to implement the various algorithms. We specifically utilized the LassoCV and GraphicalLassoCV classes from scikit-learn package [40] to implement the LASSO algorithm and estimate the precision matrix for the GGM respectively.

### Testing the prediction accuracy of different transformation methods

**Fig. 2:**
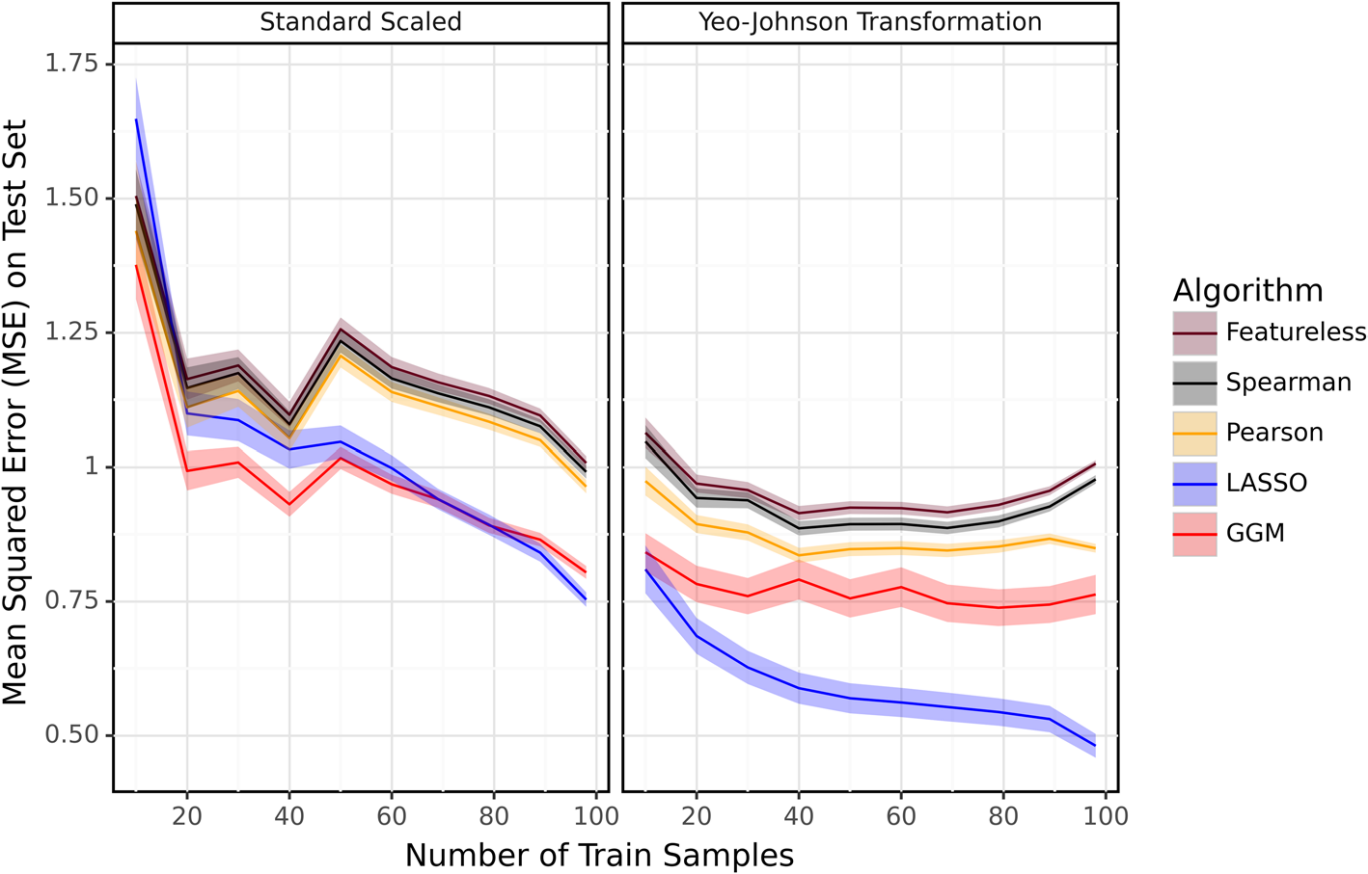
This figure evaluates the performance of Different algorithms on the Amgut2 real dataset under standard scaling only (left panel) and, Yeo-Johnson transformation and standard scaling (right panel). The results imply that just standard scaling alone (applied to the raw data set) yields lower accuracy than the combination of Yeo-Johnson and standard scaling for each of the algorithms compared

We used the Yeo-Johnson power transformation [59] in combination with standard scaling, so that each column has zero mean and unit variance (for numerical stability). The Amgut2 [24] real microbiome dataset undergoes the Yeo-Johnson transformation and standard scaling before we conduct the cross-validation analysis with various algorithms. Figure 2 shows the error on test set of the algorithms against the number of train samples. The results demonstrate that the Yeo-Johnson transformation substantially enhances the prediction accuracy on the test set relative to standard scaling only.

### Training of correlation based methods

**Fig. 3:**
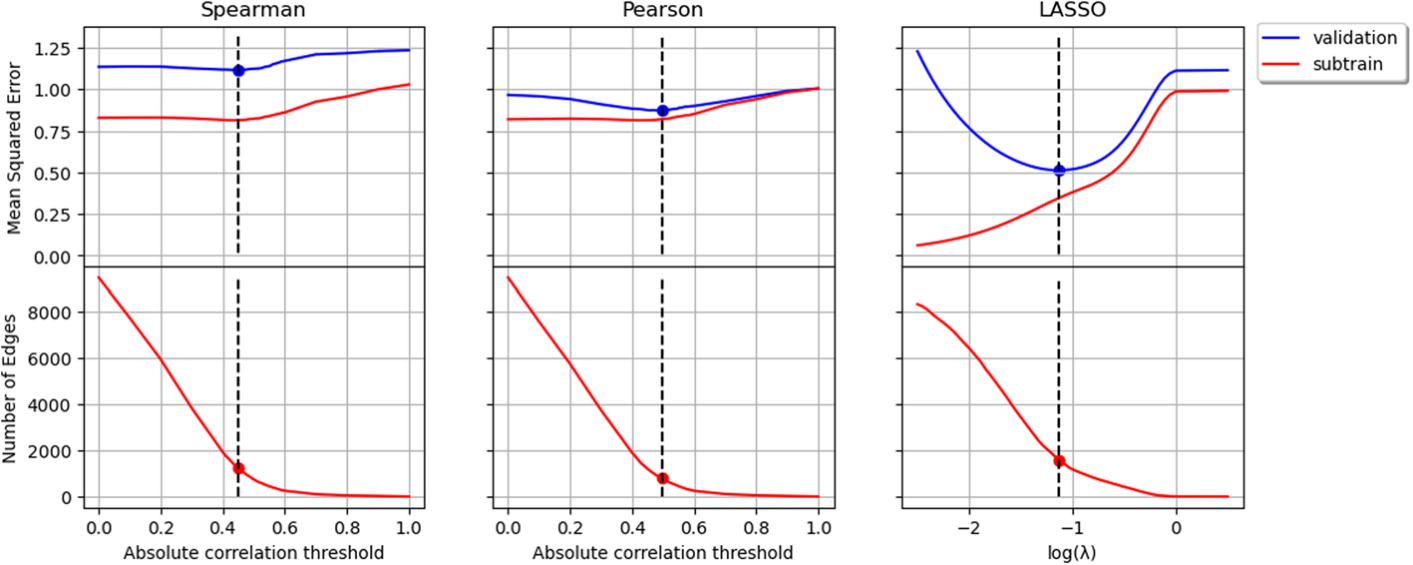
Training the Pearson correlation threshold using cross-validation

One of the most notable challenges is selecting the Pearson or Spearman’s rank correlation coefficient threshold for the co-occurrence network inference. This should be done so as to limit the network to only edges whose magnitudes are greater than the threshold. While most literature [29, 30, 41] often choose an arbitrary or pre-determined value as the correlation threshold, the choice of threshold can significantly impact the results and conclusions drawn from the analysis. Therefore, it is crucial to carefully consider and justify the choice of correlation threshold. In Figure 3, we leverage 3-fold cross-validation to choose the optimal values for the correlation coefficient threshold and $\lambda$, which minimize the validation error when used for prediction. This figure illustrates how the test error and the number of edges vary with correlation threshold for the correlation based algorithms and $\log (\lambda )$ of the LASSO model, applied to the Amgut2 data set [48]. We systematically varied these hyperparameters and monitored the resulting subtrain and validation errors. The adoption of $\log (\lambda )$, rather than $\lambda$, enhances the interpretability of the graph and mitigates potential distortion arising from extreme $\lambda$ values. The error curves reveal tendencies towards overfitting for small thresholds or $\log (\lambda )$ values (leading to many edges) and underfitting for large thresholds or $\log (\lambda )$ values (resulting in fewer edges). We selected the value of $\lambda$ corresponding to the minimum validation error, which yielded a network with 1585 edges. For the Pearson correlation coefficient, the optimal threshold was found to be 0.495, resulting in 785 edges, while for the Spearman correlation coefficient, the optimal threshold was 0.448, resulting in 1231 edges. These thresholds were chosen because they minimized the validation error, rendering correlation values smaller than these thresholds incapable of establishing edges in the co-occurrence network.

### Impact of total sample size on test error

**Fig. 4:**
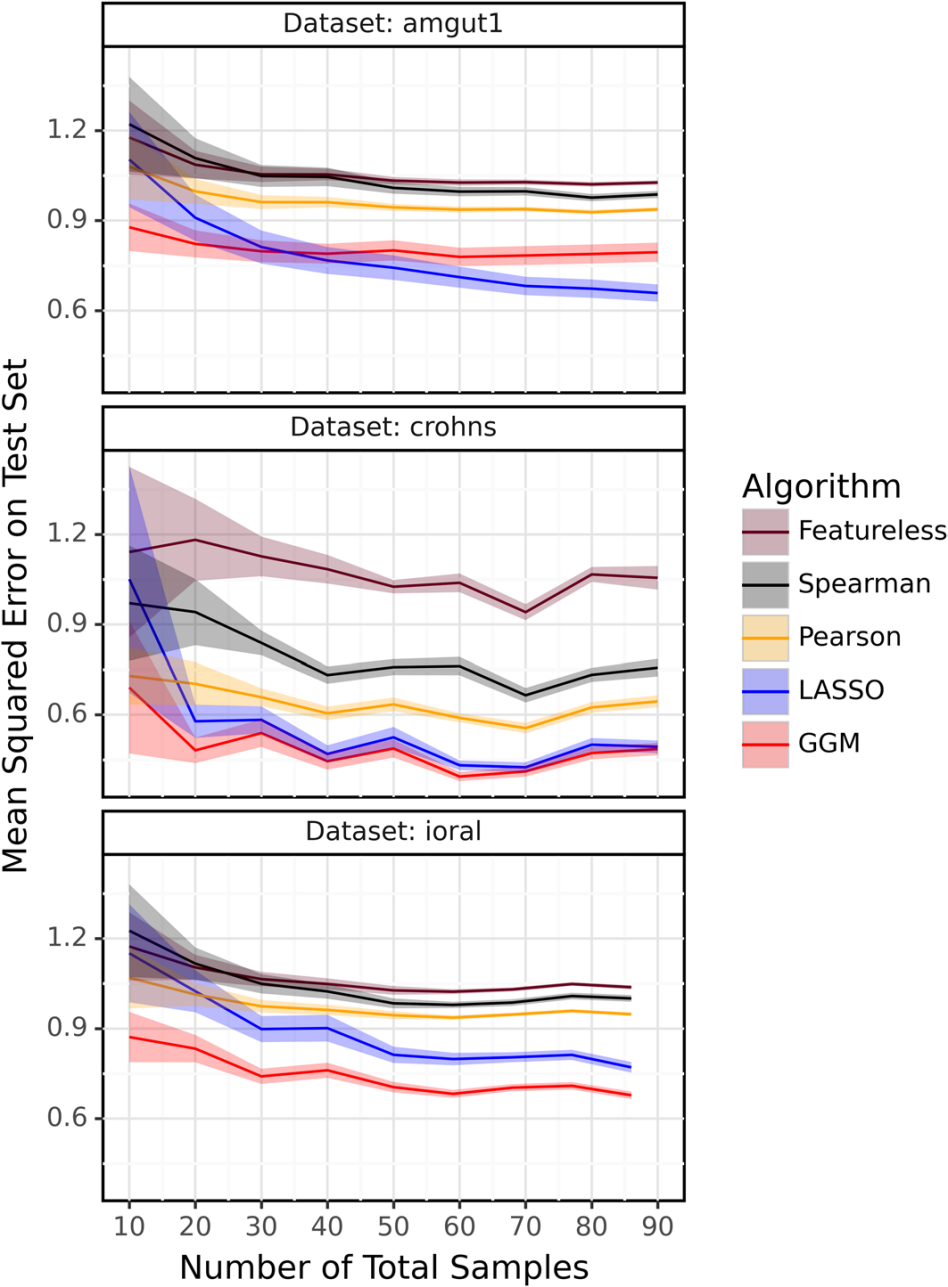
Model comparison using test error

In Figure 4, we investigated how the test error varies with the number of total samples for the various algorithms, including the featureless/baseline method, where the predicted values were computed using the mean of the train data. We sub-sample each data set by randomly dividing it into a series of different sample sizes (10, 20, etc), before we run the cross-validation analysis on each sample size. The relationship between each pair of taxon columns is utilized for prediction, as shown in the equations 1 and 2 for Pearson and Spearman algorithms respectively. The predicted value for both LASSO and GGM algorithms is calculated using the equations 3 and 4 respectively. The test error was computed by taking the average of the Mean Squared Error (MSE) of the predicted values compared to the actual test values, across all the taxa in each of the data sets, test sets and sub-samples. The lower and upper bounds of the MSE line represent the variance of the MSE. For the Amgut1 data set, GGM achieved the highest accuracy from 10 to 20 sample size, but LASSO performed best for larger sample sizes (above 30). The GGM outperformed the other algorithms on the iOral data set. The results from the crohns data set suggest that both LASSO and GGM algorithms may be good choices for this data set, as they performed similarly well. The figure also provides insights into the minimum sample size required for a useful cross-validation of the algorithms. The plot reveals that significant differences between algorithms are apparent even with only 10 samples. It is widely recognized that increasing the number of samples generally enhances correlation accuracy. However, our analysis extends this understanding by addressing a critical question: “What is the minimum number of samples required for the cross-validation technique to be effective?”. Our findings indicate that when the sample size exceeds 20 to 30, further increases in sample numbers do not significantly improve prediction accuracy. This insight is particularly valuable in the field of microbiome research, where obtaining samples is both difficult and costly. Knowing the precise sample size needed for meaningful analysis provides a novel and practical contribution to the field. These findings highlight the importance of selecting an appropriate algorithm for a given dataset, as different algorithms may perform differently depending on the characteristics of the data. Therefore, it is crucial to consider multiple algorithms and evaluate their performance before selecting the most appropriate one. In addition, it may be necessary to use a combination of algorithms to obtain the best results. In the Analysis of High-Sparsity Datasets section, we show that even highly sparse datasets do not really affect algorithm performance.

### Edge detection variability

The microbial association network was inferred using the various algorithms on the three real microbiome datasets: amgut1, crohns, and ioral.

**Fig. 5:**
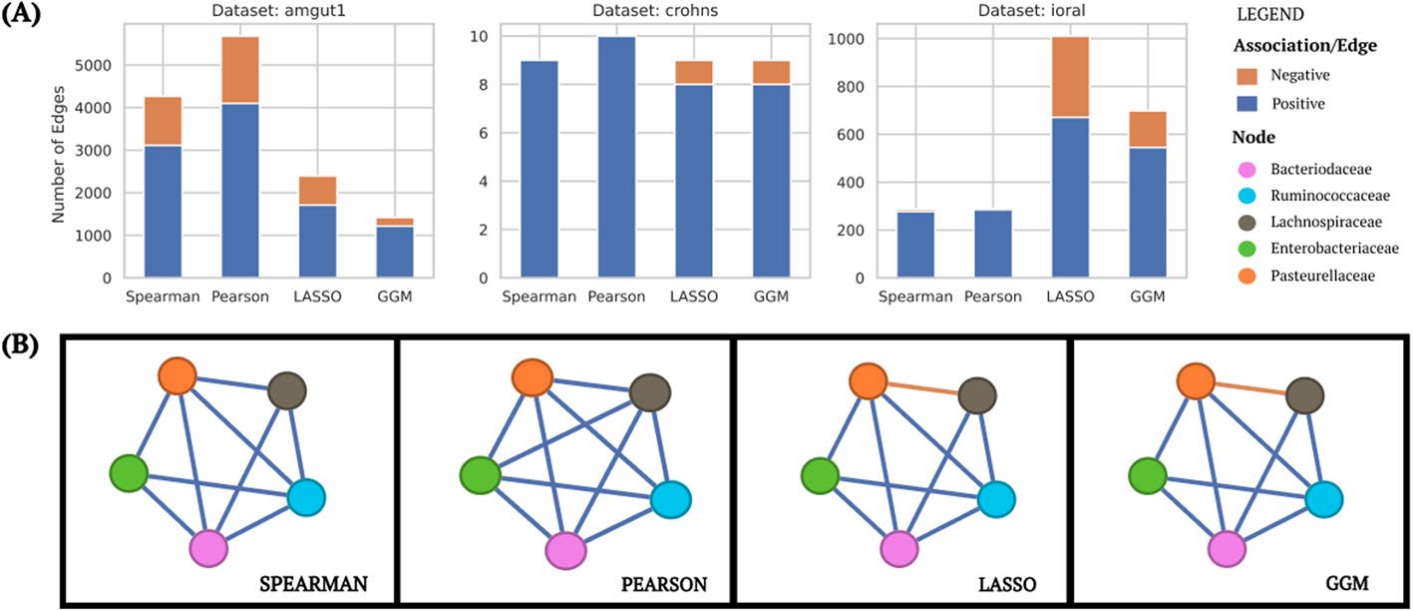
**A** Model comparison using inferred positive and negative associations. **B** Microbial Network graph of crohns data set

In Figure 5A, we showcase how the number of edges inferred varies with each of the algorithms in the various real microbiome data sets. For instance, in the amgut1 dataset, Pearson and Spearman correlation methods exhibit a significant difference in edge detection, with Pearson identifying more edges due to its sensitivity to linear relationships, whereas Spearman is better suited to non-linear associations. This pattern, however, reverses in the ioral dataset, where GGM and LASSO exhibit contrasting behavior, with LASSO producing more edges than GGM. These discrepancies highlight the importance of considering dataset-specific characteristics when selecting an algorithm for microbial network inference. Another point of interest is the presence or absence of negative links in the inferred networks, which varies significantly across algorithms. LASSO and GGM, for instance, tend to reveal negative associations that are often overlooked by correlation-based methods like Pearson and Spearman. This ability to detect negative links is crucial in understanding inhibitory interactions within the microbial community, which may have important biological implications, particularly in disease contexts such as Crohn’s disease. LASSO typically infers more number of edges than GGM because GGM employs the precision matrix that measures the partial correlation between taxa.

In Figure 5B, we present the co-occurrence network graphs of the Crohn’s disease (CD) dataset, which comprises 5 distinct bacterial taxa across 100 total samples. This simplified network reveals crucial differences in how each algorithm interprets microbial associations in the context of CD. The Pearson algorithm produces a fully connected network, suggesting complex interplay among all bacterial groups. This comprehensive view captures both strong and weak associations, potentially overestimating biologically relevant interactions. In contrast, the Spearman algorithm excludes the edge between *Lachnospiraceae* and *Enterobacteriaceae*, indicating a non-linear or rank-based relationship that Pearson’s linear correlation might overlook. Interestingly, both correlation-based methods show a strong positive association between *Bacteroidaceae* and *Enterobacteriaceae*, aligning with previous studies that suggest their co-occurrence in inflammatory environments characteristic of CD [66]. Additionally, *Lachnospiraceae* exhibits positive correlations with most other taxa in these models, reflecting its role as a core commensal group in the gut microbiome [67]. The LASSO and GGM algorithms share the same network topology as Spearman but differ significantly in the nature of the associations. Notably, both reveal a negative association between *Pasteurellaceae* and *Lachnospiraceae*, which is not captured by the correlation-based methods. This inverse relationship could indicate a potential protective role of *Lachnospiraceae* against the pro-inflammatory effects associated with some *Pasteurellaceae* species in CD [68]. The absence of an edge between *Lachnospiraceae* and *Enterobacteriaceae* in these regularized models suggests that their co-occurrence might be indirect or mediated by factors not captured in this dataset. The choice of algorithm depends critically on the research question and the data quality. If the goal is to identify a robust network structure with high confidence, prioritizing the strongest and potentially most relevant associations, then LASSO or GGM may be preferable. These methods offer a more conservative approach, effectively filtering out weaker connections that might arise from noise or indirect associations. They are particularly useful when seeking to identify key microbial players and their primary interactions in CD. However, if the research aims to explore a comprehensive and diverse network structure, including potential weak but biologically relevant associations, then Pearson or Spearman correlations might be more suitable. These methods provide a more inclusive view of all possible associations, which can be valuable for exploratory analysis and hypothesis generation. They may capture subtle relationships that could be biologically significant, especially in complex ecosystems like the gut microbiome in CD.

#### Network visualization of the american gut project 1 dataset

**Fig. 6:**
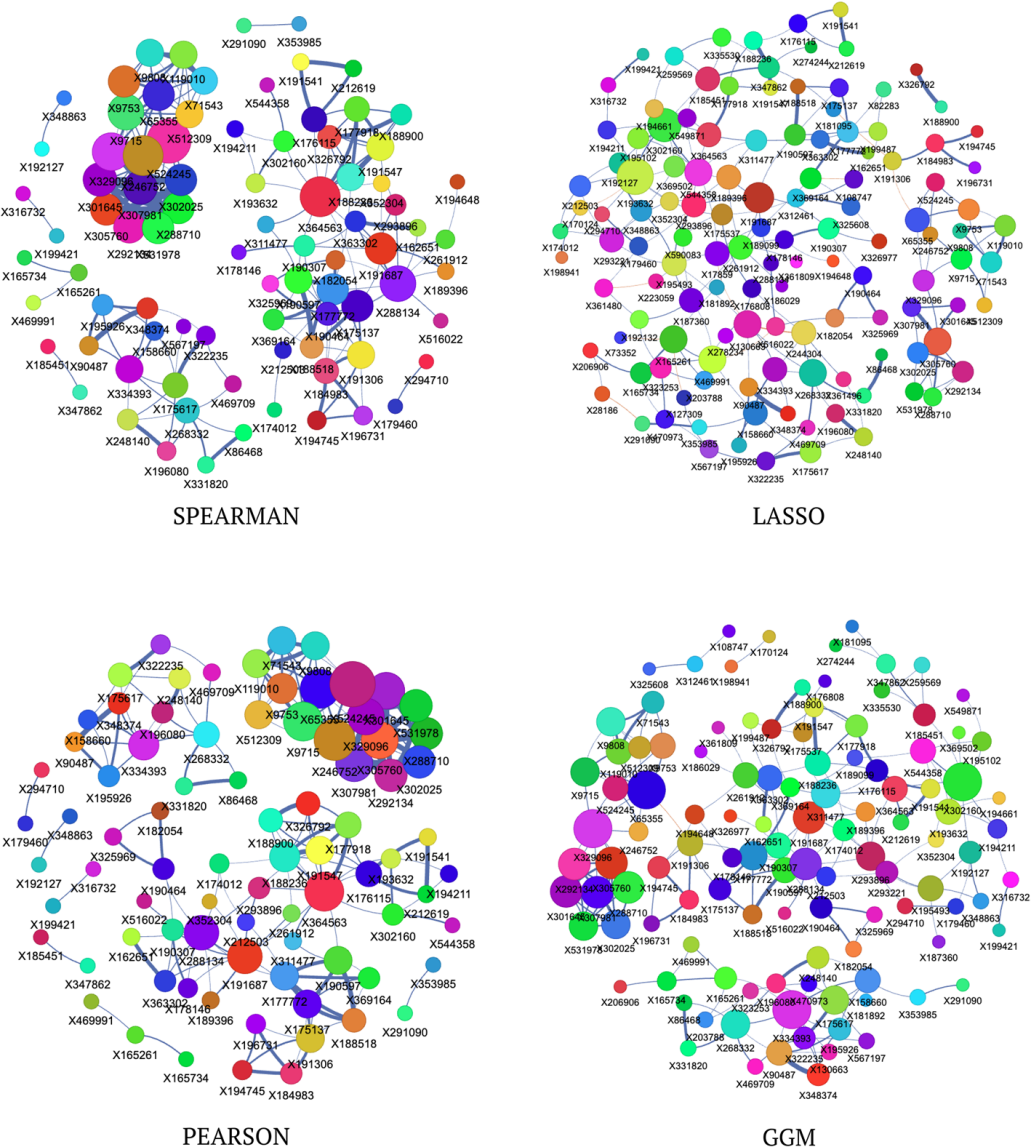
Network visualizations of the American Gut Project 1 (amgut1) dataset, showing the 200 most heavily weighted edges as determined by four different network inference algorithms: Spearman, LASSO, Pearson, and Graphical Gaussian Models (GGM)

Figure 6 illustrates network representations of the American Gut Project 1 dataset, showing the 200 strongest edges inferred by four algorithms: Spearman, LASSO, Pearson, and GGM. The Spearman network features a dense core with hubs like X197556 and X193832, alongside smaller peripheral clusters. LASSO reveals a sparser structure, emphasizing modularity with prominent clusters, such as X192161, X193832, and X188900, indicating direct relationships. The Pearson network exhibits similar density to Spearman but shifts in hub centrality, with X190649 gaining prominence over X197556. GGM emphasizes modular organization, isolating distinct clusters like X193880, X197766, and X196564, suggesting tightly linked taxa groups.

Across all methods, nodes such as X197556 and X193832 consistently serve as major hubs, underscoring their potential ecological significance in the gut microbiome. Variations in edge thickness and node size reflect differences in algorithmic inference, with LASSO capturing strong direct associations and GGM highlighting modularity.

#### Network visualization of the ioral dataset

**Fig. 7:**
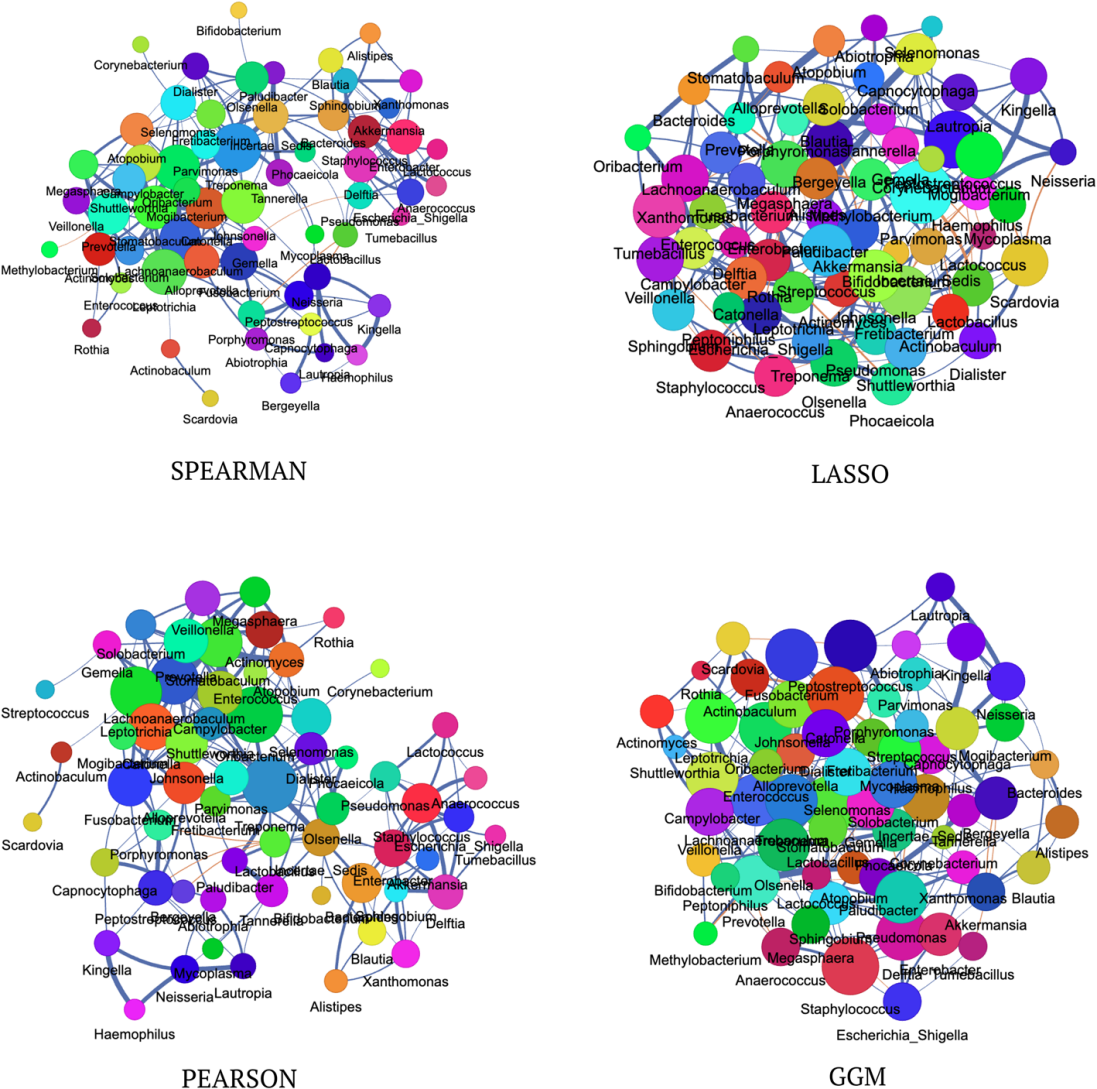
Network visualizations of the ioral dataset, showing the 200 most heavily weighted edges as determined by four different network inference methods: Spearman, LASSO, Pearson, and Graphical Gaussian Models (GGM)

Figure 7 visualizes the ioral dataset, highlighting the 200 strongest edges inferred by four network inference algorithms: Spearman, LASSO, Pearson, and GGM. The Spearman network displays a densely connected structure with Streptococcus and Veillonella as major hubs and a peripheral cluster involving Corynebacterium, Rothia, and Actinomyces, suggesting potential functional associations. LASSO reveals a sparser topology with modular clusters, including a strong Prevotella-Veillonella connection and a distinct group of Neisseria, Haemophilus, and Porphyromonas. The Pearson network, while similar in density to Spearman, highlights stronger connections for Fusobacterium and an intensified relationship between Streptococcus and Veillonella. GGM emphasizes modularity, isolating the Corynebacterium-Rothia-Actinomyces cluster, indicating its functional distinction from the broader network. Key genera, such as Streptococcus, Veillonella, and Prevotella, consistently emerge as hubs across all networks, underscoring their central ecological roles in the oral microbiome. Algorithm-specific patterns, such as the prominent Prevotella-Veillonella link in LASSO and the modularity captured by GGM, offer complementary insights into microbial relationships. The centrality of Streptococcus and Veillonella across all methods highlights their importance as keystone taxa in the oral microbial community.

## Conclusion

This study provides a comparative analysis of different algorithms for inferring microbial association networks from real microbiome composition data. We propose cross-validation as a more widely applicable evaluation criterion for training and testing various algorithms used for inferring microbial co-occurrence networks. We also introduce a novel technique of using previous algorithms for prediction on test data.

### Key Findings

Our cross-validation framework represents a methodological advance over traditional evaluation approaches like network consistency analysis. While consistency across subsamples is valuable, it can reward methods that produce overly sparse networks. In contrast, our prediction-based evaluation ensures that inferred relationships are both stable and biologically meaningful, as demonstrated by their ability to generalize to unseen data. Our study yielded several important findings:

- While it is generally recognized that algorithm accuracy improves with an increasing number of samples, our analysis identifies a threshold, beyond which additional samples offer diminishing returns. As demonstrated in Figure 4, prediction accuracy plateaus when the sample size exceeds 20–30. This finding is particularly significant in the microbiome field, where obtaining samples is both costly and difficult, as it provides valuable guidance on the minimum sample size required for effective cross-validation.
- The selection of an algorithm should depend on the specific dataset being examined and the research question being addressed, as the choice of algorithm can significantly impact the structure of the resulting microbial network.
- LASSO and GGM demonstrated the highest accuracy for inferring co-occurrence networks in the Amgut1, crohns, and iOral real microbiome composition datasets that we examined.
- Our proposed cross-validation method proved effective for both training (e.g., selecting optimal correlation thresholds) and testing the performance of various network inference algorithms.
- The Yeo-Johnson transformation, combined with standard scaling, substantially enhanced the prediction accuracy on the test set compared to standard scaling alone.

These findings highlight the importance of careful algorithm selection and data preprocessing in microbial network inference studies.

### Future directions

For future research, we are considering several avenues:

- Using equation (4) for training GGMs as well as testing, which we expect to be more accurate than employing the maximum likelihood approach to estimate the precision matrix.
- Generalizing our proposed cross-validation methods to more complex data with several qSIP features like the abundance, growth rate, death rate, and carbon uptake of micro-organisms [69].
- Exploring deep learning approaches for network inference [70]. Neural networks, particularly graph neural networks (GNNs) [71], could be adapted to learn from both abundance data and network structure simultaneously. Our cross-validation framework could be extended to evaluate these approaches, addressing challenges of limited sample sizes and high dimensionality.
- Our proposed cross-validation method, designed for microbial association network inference, has broader implications for various applications in bioinformatics. Although our current work focuses on taxa as nodes in the network, where associations are inferred based on microbial data, the same principles and algorithms could be effectively applied to other contexts where entities are represented as nodes such as:
  - Drug repurposing: Our method could potentially improve the validation of computational drug repurposing models, as discussed by Thafar et al. [72]. In this context, drugs would replace taxa as nodes, and the validation of drug repurposing models would benefit from our rigorous cross-validation strategy. This could lead to more reliable predictions of potential new uses for existing drugs.
  - Drug-drug interaction prediction: The cross-validation approach might enhance the performance evaluation of models predicting drug-drug interactions, similar to the work by Feng et al. [73]. This could contribute to improved patient safety and more effective combination therapies.
  - RNA N6-Methyladenosine modification site prediction: Our method could be adapted to improve the validation of models predicting RNA modification sites, as explored by Fu et al. [74]. This could advance our understanding of post-transcriptional regulation mechanisms.

The potential applications of our cross-validation scheme in these areas underscore the broad relevance of our work beyond microbial network inference. By providing a more robust evaluation framework, our method could contribute to increased reliability and reproducibility across various bioinformatics domains. In conclusion, this study demonstrates the effectiveness of cross-validation for evaluating and comparing microbial network inference algorithms. Our findings indicate that both LASSO and GGM are dependable and effective for inferring co-occurrence networks. The proposed methodology not only improves the reliability of microbial network inference but also has potential applications in other areas of bioinformatics and computational biology. Future work will focus on refining these methods and exploring their broader applicability in the field.

## Additional file

Supplementary file 1.

## Data Availability

The data and code used in this study are publicly available at https://github.com/EngineerDanny/Microbe-Network-Research
