# Supplementary material for: Cross-validation for training and testing co-occurrence network inference algorithms
Source: BMC Bioinformatics. 2025 Mar 6;26:74. doi: 10.1186/s12859-025-06083-7 (PMC11883995; doi:10.1186/s12859-025-06083-7)
Supplement: Supplementary file 1 [file 12859_2025_6083_MOESM1_ESM.pdf]

Supporting Information

Gaussian Graphical Model Proof Let D be the total number of taxa, $\boldsymbol{x}$ be the D-dimensional row/sample vector, which we assume follows a multivariate normal distribution with mean 0 and $D \times D$ covariance matrix, $\boldsymbol{\Sigma}$ of size $D \times D$. Let $\boldsymbol{\Omega}$ be the $D \times D$ precision matrix comprised of ω_{ij} elements and $\boldsymbol{x}^T$ denote the transpose of $\boldsymbol{x}$.

Proposition 1. *Equation (4) can be used to compute the conditional mean of one taxon x_1 in a Gaussian Graphical Model, given the other taxa $x_2, \ldots, x_D$.*

Proof. The multivariate normal distribution Probability Density Function is given by:

$$f(\boldsymbol{x}) = \frac{1}{\sqrt{(2\pi)^D|\boldsymbol{\Sigma}|}} \exp\left(-\frac{1}{2}\boldsymbol{x}^T\boldsymbol{\Omega}\boldsymbol{x}\right)$$

The predicted value of taxon x_1 can be calculated by finding the conditional mean of the distribution via the equation below.

$$\begin{aligned}
\frac{\partial}{\partial x_1} f(\boldsymbol{x}) &= 0 \\
\frac{\partial}{\partial x_1}\left(\frac{1}{\sqrt{(2\pi)^D|\boldsymbol{\Sigma}|}} \exp\left(-\frac{1}{2}\boldsymbol{x}^T\boldsymbol{\Omega}\boldsymbol{x}\right)\right) &= && \text{(Substitute } f(\boldsymbol{x}) \text{)} \\
\frac{\partial}{\partial x_1} \exp\left(-\frac{1}{2}\boldsymbol{x}^T\boldsymbol{\Omega}\boldsymbol{x}\right) &= && \text{(Remove constants)} \\
\exp\left(-\frac{1}{2}\boldsymbol{x}^T\boldsymbol{\Omega}\boldsymbol{x}\right)\frac{\partial}{\partial x_1}\left(-\frac{1}{2}\boldsymbol{x}^T\boldsymbol{\Omega}\boldsymbol{x}\right) &= && \text{(Differentiate expression)} \\
\frac{\partial}{\partial x_1}\left(\boldsymbol{x}^T\boldsymbol{\Omega}\boldsymbol{x}\right) &= && \text{(Remove constants and simplify)} \\
\frac{\partial}{\partial x_1}\left(\sum_{i=1}^{D}\sum_{j=1}^{D}\omega_{ij}x_ix_j\right) &= && \text{(Expand the bracket)} \\
\frac{\partial}{\partial x_1}\left(\omega_{11}x_1^2 + \sum_{i=2}^{D}\omega_{i1}x_ix_1 + \sum_{j=2}^{D}\omega_{1j}x_1x_j\right) &= && \text{(Simplify further)} \\
2\omega_{11}x_1 + \sum_{i=2}^{D}\omega_{i1}x_i + \sum_{j=2}^{D}\omega_{1j}x_j &= 0 && \text{(Differentiate expression)} \\
\implies \frac{-1}{2\omega_{11}}\left(\sum_{i=2}^{D}\omega_{i1}x_i + \sum_{j=2}^{D}\omega_{1j}x_j\right) &= x_1 && \text{(Solve for } x_1 \text{)}
\end{aligned}$$

□

Corollary 1. *For the special case of $D = 2$, the multivariate Gaussian conditional mean (4) simplifies to (1) under the assumption that the data is standard scaled; zero mean and unit variance for each taxon.*

Proof. For a pair (x_1, x_2) of taxa that follow a bivariate normal distribution with correlation coefficient ρ_{x_1,x_2},

marginal standard deviations σ_{x_1} and σ_{x_2}, the predicted value of x_1 given x_2 is given below.

$$\mathbf{\Omega} = \frac{1}{(1-\rho^2_{x_1,x_2})}\begin{bmatrix} \frac{\sigma^2_{x_2}}{\sigma^2_{x_1}\sigma^2_{x_2}} & -\frac{\rho_{x_1,x_2}}{\sigma_{x_1}\sigma_{x_2}} \\ -\frac{\rho_{x_1,x_2}}{\sigma_{x_1}\sigma_{x_2}} & \frac{\sigma^2_{x_1}}{\sigma^2_{x_1}\sigma^2_{x_2}} \end{bmatrix} \qquad (\text{ Define } \mathbf{\Omega}\text{, when } D=2 \text{ })$$

$$= \begin{bmatrix} \frac{1}{\sigma^2_{x_1}(1-\rho^2_{x_1,x_2})} & -\frac{\rho_{x_1,x_2}}{\sigma_{x_1}\sigma_{x_2}(1-\rho^2_{x_1,x_2})} \\ -\frac{\rho_{x_1,x_2}}{\sigma_{x_1}\sigma_{x_2}(1-\rho^2_{x_1,x_2})} & \frac{1}{\sigma^2_{x_2}(1-\rho^2_{x_1,x_2})} \end{bmatrix} \qquad (\text{ Expand and simplify })$$

$$x_1 = \frac{-1}{2\omega_{11}}\left(\sum_{i=2}^{2}\omega_{i1}x_i + \sum_{j=2}^{2}\omega_{1j}x_j\right) \qquad (\text{ Equation for } x_1\text{, when } D=2 \text{ })$$

$$= \frac{-1}{2\omega_{11}}(\omega_{21}x_2 + \omega_{12}x_2) \qquad (\text{ Simplify })$$

$$= \frac{-1}{\omega_{11}}(\omega_{21}x_2) \qquad (\text{ Substitute } \omega_{21} = \omega_{12} \text{ from } \mathbf{\Omega} \text{ })$$

$$= \frac{-1}{\left(\frac{1}{\sigma^2_{x_1}(1-\rho^2_{x_1,x_2})}\right)}\left(-\frac{\rho_{x_1,x_2}}{\sigma_{x_1}\sigma_{x_2}(1-\rho^2_{x_1,x_2})}x_2\right) \qquad (\text{ Substitute the values of } \omega \text{ })$$

$$\implies x_1 = \rho_{x_1,x_2}\frac{\sigma_{x_1}}{\sigma_{x_2}}(x_2) \qquad (\text{ Simplify })$$

□

Analysis of High-Sparsity Datasets To address the influence of higher sparsity levels on algorithm performance, we analyzed two additional real datasets: glne007 (58.88% sparse) and mixmpln (69.82% sparse). As shown in Figure 8, GGM maintains robust performance across both high-sparsity datasets, while LASSO's performance improves with increasing sample size. In the glne007 dataset, GGM consistently outperforms other methods, suggesting it handles sparse data well. The mixmpln dataset shows similar trends, with both GGM and LASSO eventually achieving better prediction accuracy than correlation-based methods as sample size increases. These results support our original findings while demonstrating that our conclusions about algorithm performance hold even for datasets with higher sparsity levels.

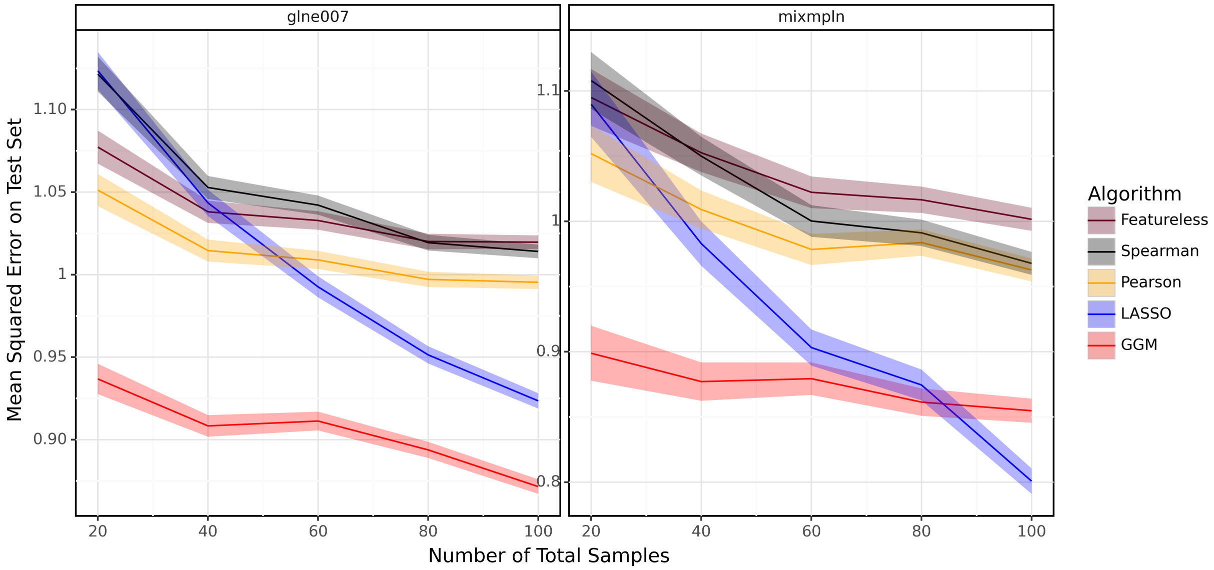

Figure 8: Performance comparison of different algorithms on high-sparsity datasets.
